# From Mandatory to Meaningful: Boosting Engagement With Announced Bonus Quizzes

**DOI:** 10.1002/jdd.70096

**Published:** 2025-10-27

**Authors:** Philip Patston, Anthony Huang, Asha Eapen

**Affiliations:** ^1^ Department of Oral Medicine and Diagnostic Sciences, College of Dentistry University of Illinois Chicago Chicago Illinois USA

## 1 Problem

First‐year dental students at UIC College of Dentistry face a demanding academic load in their biomedical science courses. They must manage large volumes of complex material, adjust to independent learning, and prepare for high‐stakes exams early in their dental education. Although attendance for certain sessions is mandatory, maintaining consistent student engagement has been challenging. Some students opt to review content independently, perceiving live sessions as less beneficial than self‐paced study. This aligns with research suggesting that students' motivation and learning strategies are often influenced by their perceived value of instructional activities [1]. To address both the academic and motivational challenges, announced bonus quizzes were introduced as a strategic intervention. These quizzes served not only as a content reinforcement tool but also as a tangible incentive for students to attend class, actively participate, and stay engaged with the curriculum [2].

## 2 Solution

**FIGURE 1:**
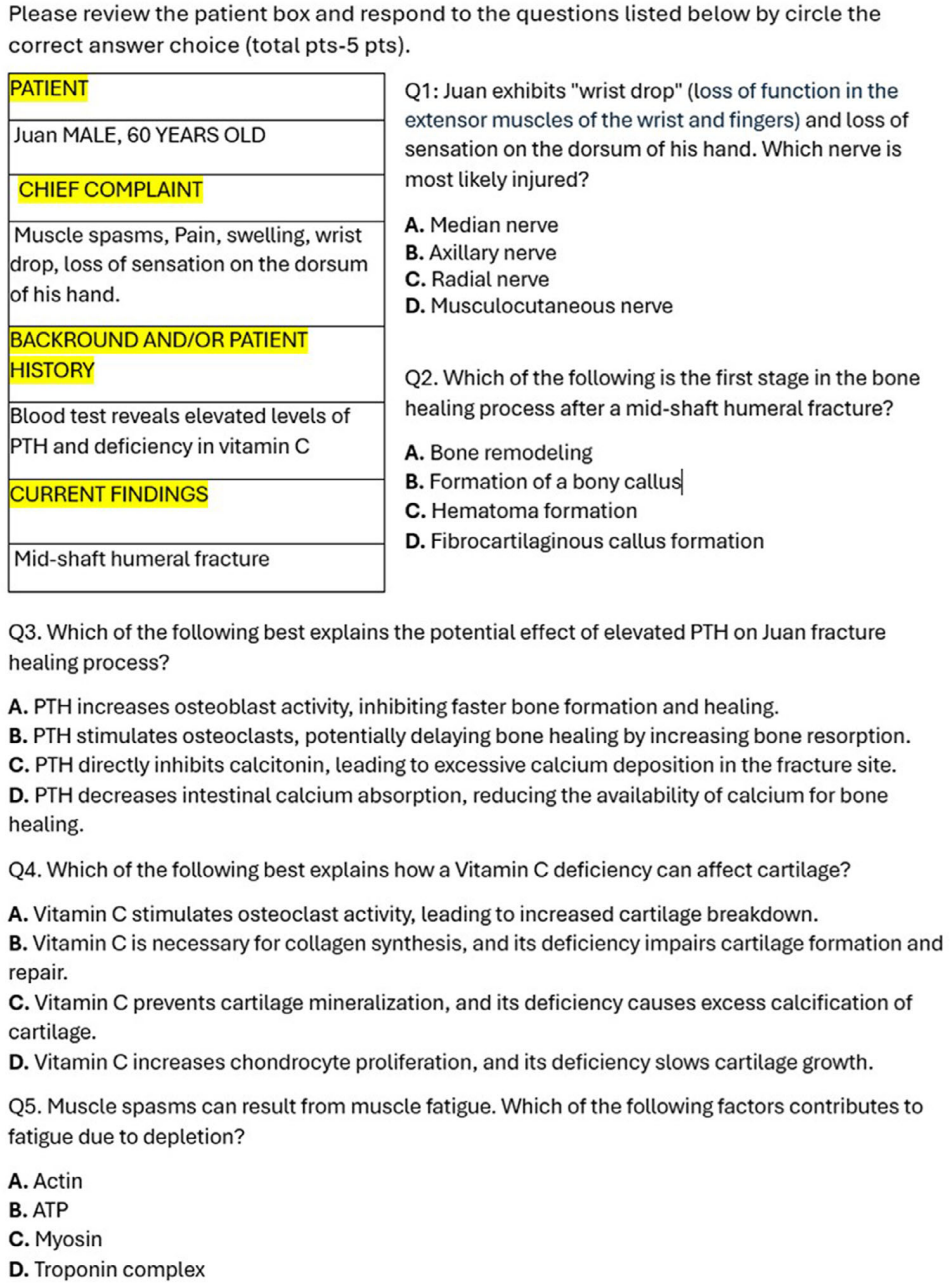
Patient Box with Bonus Quizzes. The first‐year dental curriculum follows an integrated structure with seven modules (three fall, four spring), each concluding with a 90‐minute in‐person Q&A session held the day before module exams. During the first 15–20 minutes of each session, announced bonus quizzes were administered and immediately reviewed to reinforce key concepts and provide feedback. These quizzes consisted of five multiple‐choice questions in INDBE format using patient‐based clinical scenarios to mirror board‐style examinations, with each quiz worth up to 5 bonus points applicable to the corresponding module exam, serving as a low‐stakes motivational tool to encourage engagement and preparation. The activity was conducted as part of normal course delivery and framed as an educational quality improvement initiative to enhance engagement in mandatory Q&A sessions.

To support learning and boost in‐person engagement, announced bonus quizzes were implemented during the Q&A session preceding each biomedical module exam. Each quiz included five multiple‐choice questions designed in the INDBE format, using patient box clinical scenarios to mirror board‐style exams (Figure 1). While attendance at these Q&A sessions was expected, the addition of a bonus quiz created a meaningful reason for students to show up and participate. The opportunity to earn bonus points provided a low‐pressure, high‐reward environment that encouraged preparation and focus—an approach supported by studies showing the benefits of low‐stakes, test‐enhanced learning [3]. Because the quizzes were announced in advance, students could study intentionally and walk into the session feeling more confident. Reviewing the quizzes together further reinforced critical content and provided immediate feedback, strengthening the connection between classroom learning and clinical application [4]. To deepen the incentive, one of the quiz questions was later included on the actual module exam as a bonus question.

## 3 Results

**FIGURE 2:**
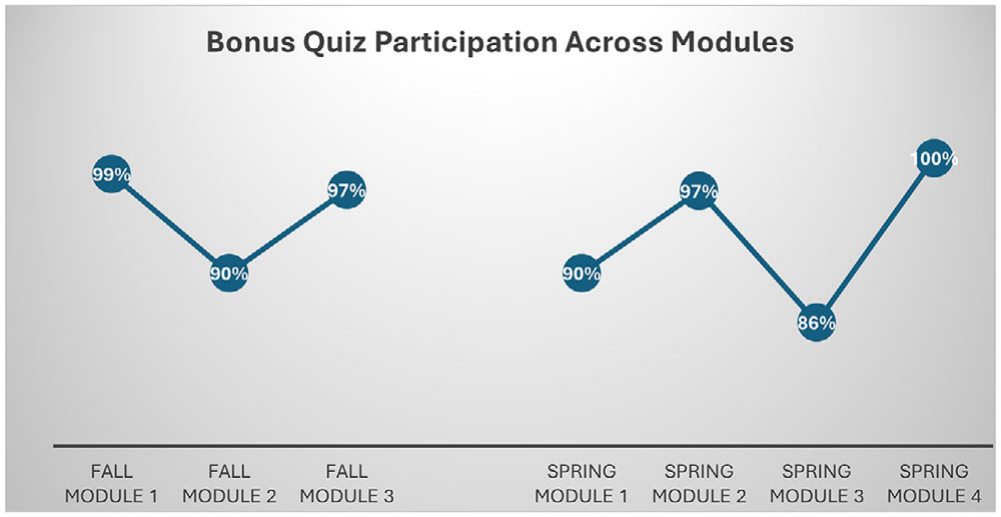
**Bonus Quiz Participation Across Modules**. Our study compared attendance data between two consecutive cohorts: the Class of 2027 (*n* = 70 students, pre‐intervention) and the Class of 2028 (*n* = 72 students (fall semester) and *n* = 70 (spring semester), post‐intervention). Attendance was calculated as the percentage of enrolled students present during each of the seven annual Q&A sessions (one per module), representing targeted intervention points where engagement was most challenging despite mandatory attendance policies. For the Class of 2027, attendance data were drawn from faculty records of Q&A session participation, while for the Class of 2028, attendance was documented through quiz participation records since physical presence was required to complete the bonus quiz. This line graph illustrates student participation across all biomedical modules for both semesters, showing a consistent increase in attendance for the Class of 2028 compared to the Class of 2027, where attendance remained below 50% for each Q&A session, demonstrating that the introduction of announced bonus quizzes served as an effective motivational tool throughout the academic year.

Although attendance was required, many students in the Class of 2027 (prior to quiz implementation) still opted not to attend, with attendance falling below 50% across both semesters. This highlights the limitations of policy‐based attendance without meaningful incentives or consequences. In contrast, after implementing bonus quizzes for the Class of 2028, attendance rose substantially from 90.0% in Fall Module 1% to 100% by the end of spring (Figure 2). These gains suggest that the opportunity to earn bonus points served as a strong motivator for students to attend and engage in sessions that were previously underutilized. Based on instructor observations, the announced nature of the quizzes appeared to encourage students to study more intentionally and arrive better prepared. The low‐stakes format reinforced key concepts, reduced anxiety, and gave students greater control over their performance. Faculty observations supported these outcomes, noting improved attentiveness, discussion, and quality of questions. Although formal exam score comparisons were not conducted, the shift in attendance, paired with positive feedback, strongly supports the effectiveness of announced bonus quizzes. Over time, the structured rhythm of these assessments fostered consistent study habits and a deeper connection with the curriculum.
